# Effect of Ozonated Water in Nonsurgical Management of Periodontitis

**DOI:** 10.1002/cre2.70216

**Published:** 2025-09-08

**Authors:** Mohadese Yazdanpanahbahabadi, Mojan Ghafouri, Majid Fereidooni, Hamed Hosseinkazemi

**Affiliations:** ^1^ Department of Periodontology Babol University of Medical Sciences Babol Mazandran Iran; ^2^ Department of Periodontology Babol University of Medical Sciences Babol Mazandaran Iran; ^3^ Department of Oral Medicine Babol University of Medical Sciences Babol Mazandaran Iran

**Keywords:** chronic periodontitis, ozone, periodontal debridement

## Abstract

**Objectives:**

Due to ozonated water's antimicrobial and anti‐inflammatory properties, it may be used as a supplementary treatment to scaling and root planing (SRP). The present study aimed to evaluate the clinical effectiveness of using ozonated water instead of tap water during the SRP for people with chronic generalized periodontitis.

**Material and Methods:**

This randomized clinical trial was conducted on 30 patients with generalized periodontitis Stages I and II, randomly allocated to two groups. Test sites were subjected to ozonated water, and control sites were subjected to tap water. The clinical periodontal parameters were measured at baseline, the 4th, and 8th week of treatment.

**Results:**

Findings showed a significant decrease in periodontal pocket depth (PPD), clinical attachment level (CAL), and gingival index (GI) at both the 4th and the 8th week of treatment in both groups. PPD was further reduced in the test group, but the difference between the two groups was not significant. However, the greater reduction of CAL at the 4th and 8th week (*p* < 0.001 and *p* < 0.001, respectively) and also the greater reduction of GI at the 4th week (*p* = 0.01) was observed in the test group.

**Conclusions:**

Both the test and control groups showed clinical improvement following nonsurgical periodontal therapy. The ozonated water group demonstrated significantly greater reductions in CAL at the 4th and 8th weeks, and in GI at the 4th week. However, the magnitude of these differences was modest and may have limited clinical relevance. Further research is needed to confirm long‐term outcomes.

## 1 Introduction

Periodontal disease is a bacterial infection where the tissues and bones supporting the teeth are destroyed (Listgarten 1987; Slots 1986). Microorganisms are primarily responsible for these destructive inflammatory responses. Porphyromonas gingivalis is a type of anaerobic bacteria commonly found in deep periodontal pockets. The primary purpose of periodontal treatments is the removal of bacterial plaque.

The prevalence and severity of periodontal diseases are managed by reducing bacterial plaque or, at least, pathogenic bacteria (Goodman and Robinson 1990). Scaling and root planing (SRP) is considered the most common nonsurgical periodontal treatment. This treatment cannot completely eliminate periodontal pathogens, particularly in deep areas of the pocket, furcations, interproximal spaces, and root concavities (Plessas 2014).

The effectiveness of SRP differs depending on the access to the depth of the pocket, leading to the use of supplementary antibacterial agents in the form of irrigants (Canadian Agency for Drugs and Technologies in Health 2016). Studies conducted in the past two decades have reported improvements in clinical parameters with the use of irrigants, including povidone‐iodine, hydrogen peroxide, stannous fluoride, boric acid, and chlorhexidine. However, each irrigant has its own disadvantages, which have led to its limited application (Wolff et al. 1989; Rosling et al. 1983; Schmid et al. 1985; Sağlam et al. 2013; Walsh et al. 1992). Some adverse effects such as desquamative mucositis, tooth discoloration, altered taste, and allergies (e.g., anaphylactic shock) have been reported for chlorhexidine (Walsh et al. 1992).

Moreover, povidone‐iodine is contraindicated in some individuals, including those allergic to iodine, those with thyroid dysfunction, and pregnant or lactating women (Al Habashneh et al. 2015). Therefore, using a safe antiseptic with high antimicrobial properties and few contraindications as an irrigant adjunct to SRP treatment can be beneficial (Rosling et al. 1983).

Recently, ozone (O3) therapy has become popular in dentistry. Ozone is a gas commonly present in the upper parts of the atmosphere (Lynch 2009). It has antimicrobial effects against bacteria, viruses, fungi, and protozoa by damaging their cytoplasmic membrane and altering their intracellular contents. This effect is nonspecific but selectively targets microbial cells; human body cells are not affected due to their antioxidative defenses (Seidler et al. 2008; Huth et al. 2006). Ozone also activates the cellular and humoral immune systems by stimulating the proliferation of immune cells and the synthesis of immunoglobulins. Moreover, from a biological standpoint, it activates the synthesis of substances such as interleukins, leukotrienes, and prostaglandins that reduce inflammation and accelerate wound healing. Compared to sodium hypochlorite 2.5%, ozonated water has considerable antimicrobial activity. Furthermore, the metabolic activity of fibroblasts is increased in tissues treated with ozonated water (Seidler et al. 2008).

The application of ozone in dentistry includes the treatment of caries and dental hypersensitivity, root canal therapy, bleaching, mucosal lesions, periodontitis, and peri‐implantitis (Lynch 2009). Both gaseous and aquatic forms of ozone have been used in periodontal treatments, but ozonated water is easier to use (Ramzy et al. 2005). It has also been reported that aquatic forms of ozone have a high level of biocompatibility with fibroblasts, cementoblasts, and epithelial cells. Therefore, the use of ozonated water is appropriate for the treatment of oral infections, including periodontal disease (Nagayoshi et al. 2004).

Numerous studies have reported considerable improvement in clinical parameters in patients with periodontitis following SRP along with the application of ozonated water (Walsh et al. 1992; Ramzy et al. 2005; Yılmaz et al. 2013; Hayakumo et al. 2013). Additionally, Dhingra et al. conducted a pilot study on 50 orthodontic patients and reported that the use of ozonated water had a significant effect on the treatment of gingival inflammation caused by orthodontic treatments (Nogales et al. 2008). Only a limited number of studies have focused on the effect of ozonated water on chronic periodontitis, but they have reported contradictory results regarding the improvement in clinical parameters after treatment (Al Habashneh et al. 2015; Lynch 2009; Seidler et al. 2008; Huth et al. 2006; Ramzy et al. 2005; Nagayoshi et al. 2004; Nogales et al. 2008; Katti and Chava 2013; Yılmaz et al. 2013; Hayakumo et al. 2013; Kshitish and Laxman 2010; Uraz et al. 2019; Katti and Chava 2013).

Therefore, due to the antimicrobial properties of ozonated water, the present study examined the effect of using ozonated water in place of tap water during SRP treatment for patients with Stage I and II periodontitis on the further elimination of pathogens and the improvement of clinical parameters.

## 2 Materials and Methods

This double‐blind randomized clinical trial included 30 patients, aged 26–57 years, who attended the Department of Periodontology at the Faculty of Dentistry, Babol University of Medical Sciences. The sample size was calculated on the basis of previous literature (Hayakumo et al. 2013). All participants were generally healthy and diagnosed with Stage I and II periodontitis, following the criteria set by the Workshop for Classification of Periodontal and Peri‐Implant Diseases co‐sponsored by the American Academy of Periodontology (AAP) and the European Federation of Periodontology (EFP) in 2017 (Caton et al. 2018). Their O'Leary plaque index was ≤ 20. Patients were excluded if they had a history of systemic diseases that could affect C‐Reactive Protein (CRP) levels, a positive smoking history, were pregnant or lactating, had received surgical or nonsurgical periodontal treatment or antibiotics in the past 6 months, or had contraindications for the use of the ultrasonic device. The study objectives and treatment stages were thoroughly explained to all patients, who then provided informed consent. The study protocol received approval from the ethical committee of Babol University of Medical Sciences and was registered at http://www.irct.ir. The registration number for this trial is IRCT20090311001760N48, and the registration date is September 17, 2019.

### 2.1 Pretreatment stage

Initially, patients were randomly assigned using a computer‐generated table to one of two groups: one receiving SRP with tap water (control group) or with ozonated water (test group) (*n* = 15 each). This random allocation was conducted by the first clinician. Both groups were balanced in terms of age, sex, and periodontal condition. All participants received Oral Hygiene Instruction (OHI) to ensure they maintained proper oral hygiene during the treatment period and between follow‐up visits. The OHI included guidance on brushing twice daily with a soft brush using the Bass method, flossing, and, if necessary, interdental brushing. Subsequently, clinical parameters such as periodontal pocket depth (PPD), clinical attachment level (CAL), and gingival index (GI) in three specific regions (mesiobuccal, midbuccal, and distobuccal) were assessed by the second clinician, who was blinded to group assignments and had undergone formal calibration, including supervised clinical probing training, to ensure high intra‐examiner reproducibility. The following clinical parameters were measured for all teeth using a University of Michigan “O” probe with Williams marking, and the values were rounded to the nearest 0.5 mm.

1. PPD: This measures the distance from the most apical part of the gingival margin to the bottom of the periodontal pocket.
2. CAL: This indicates the distance from the cemento–enamel junction (CEJ) to the bottom of the periodontal pocket.
3. GI (Löe and Silness): This assesses the severity of gingival inflammation, evaluating parameters based on color, consistency, and bleeding on probing.

These measurements help evaluate the periodontal health and treatment effects within the study groups.

### 2.2 Treatment Phase

SRP was performed by the third clinician who was blinded to the group allocation. The SRP procedure included comprehensive debridement of both supragingival and subgingival plaque and calculus using a DTE D7 ultrasonic device. SRP was conducted over two sessions, each lasting 40 min, with a 1‐week interval between sessions.

The DTE D7 ultrasonic device is equipped with a water tank, which was filled either with tap water or ozonated water based on the treatment design determined by the first clinician.

### 2.3 Water Management

Due to the half‐life of ozone in water, the ozonated water in the tank was replaced every 20 min to maintain its efficacy (Nagayoshi et al. 2004). To ensure consistency between test and control groups, the tap water was also replaced every 20 min during the treatment phases.

These steps were taken to ensure that both treatment groups received consistent and systematic care.

### 2.4 Ozonated Water Preparation

Ozonated water was prepared using an Ozone Generator SS8 device connected to a NIDEK Oxygen Concentrator (Nuvo Lite).

• Oxygen Concentrator Specifications:
  ◦ Delivered oxygen at a flow rate of 5 L/min.
  ◦ Operated at a pressure of 7 PSI with a 95% oxygen concentration.
• Ozone Generation Process:
  ◦ The Ozone Generator converted the supplied oxygen into gaseous ozone using the Corona Discharge phenomenon (Rakness et al. 1996).
  ◦ The ozone gas was then introduced into a humidifier bottle filled with tap water through a humidifier tube, allowing the ozone to dissolve into the water through a bubbling ozonation process (Rakness et al. 1996).
• Concentration and Verification:
  ◦ Ozonated water was prepared at a concentration of 1.5 parts per million (ppm) (Nagayoshi et al. 2004).
  ◦ The ozone concentration was verified using a colored dye test to ensure consistency, with a minimum contact time of 15 min required to achieve the 1.5 ppm concentration (Nagayoshi et al. 2004).
• Water Management During Treatment:
  ◦ Approximately 500 mL of ozonated water was prepared before each SRP session.
  ◦ Due to the half‐life of ozone in water, the ozonated water in the ultrasonic device tank was replaced every 20 min to maintain effective antimicrobial activity (Nagayoshi et al. 2004).
  ◦ For consistency, in the control group, tap water was also refreshed every 20 min.

This standardized preparation ensured that the ozonated water used during the periodontal treatment maintained both the required concentration and biological activity throughout the procedure.

### 2.5 Posttreatment Stage

After SRP procedure, patients received oral hygiene instructions again. Follow‐up sessions were scheduled 4 weeks and 8 weeks after the second stage of treatment. During these visits, the second clinician examined the patients' clinical parameters using the previously described methods and recorded the measurements.

#### 2.5.1 Data Analysis Method

The data were analyzed using SPSS version 21 software. Changes in parameters over the different time intervals were assessed. The mean and standard deviation (SD) of all clinical variables were calculated for both groups.

### 2.6 Statistical Tests Used

– Independent *t*‐test for comparisons between groups.
– Repeated measures ANOVA for comparisons within groups.

A significance level of *p* < 0.05 was set for all statistical analyses to determine the significance of the findings.

## 3 Results

A total of 30 eligible patients participated in this study, divided into test and control groups. One patient from the test group was excluded due to not attending follow‐up visits, and as a result, their data were removed from the analysis. The demographic and clinical parameters, including PPD, CAL, and GI, showed no significant differences between the two groups before the treatment (*p* > 0.05) (Table 1). The mean age was 36.06 ± 8.40 years in the test group and 36.06 ± 8.48 years in the control group.

**Table 1:** Demographic information and clinical parameters of patients before treatment.

Parameter	Test group (*n* = 14) Mean ± SD	Control group (*n* = 15) Mean ± SD	*p* value
Sex, *n* (%)			
Male	4 (28.6%)	5 (33.3%)	0.78
Female	10 (71.4%)	10 (66.7%)	0.78
Age (years)	36.06 ± 8.40	36.06 ± 8.48	0.96
PPD (mm)	2.21 ± 0.67	2.16 ± 0.60	0.83
CAL (mm)	3.02 ± 0.52	3.09 ± 0.37	0.67
GI	1.66 ± 0.37	1.86 ± 0.15	0.08

The mean CAL, PPD, and GI at 4 weeks and 8 weeks for both groups are presented in Table 2. Repeated measures ANOVA indicated that mean CAL, PPD, and GI in both groups significantly decreased after 4 and 8 weeks.

**Table 2:** Mean measurement of clinical parameters before, week 4, and week 8 of the treatment.

Parameter	Time	Control group Mean ± SD	*p* value^a^	Test group Mean ± SD	*p* value^a^	*p* value^b^
PPD (mm)	Before treatment	2.16 ± 0.60	< 0.001	2.21 ± 0.67	< 0.001	0.83
	Week 4	1.88 ± 0.38		1.67 ± 0.27		0.10
	Week 8	1.74 ± 0.36		1.53 ± 0.28		0.10
CAL (mm)	Before treatment	3.09 ± 0.37	< 0.001	3.02 ± 0.52	< 0.001	0.67
	Week 4	2.75 ± 0.29		2.36 ± 0.26		< 0.001
	Week 8	2.65 ± 0.28		2.12 ± 0.39		< 0.001
GI	Before treatment	1.86 ± 0.15	< 0.001	1.66 ± 0.37	< 0.001	0.08
	Week 4	1.23 ± 0.29		1.02 ± 0.58		0.01
	Week 8	1.09 ± 0.18		1.00 ± 0.00		0.059

^a^ Within groups.

^b^ Between groups.

Although the PPD decreased more in the test group at both 4 and 8 weeks, the differences were not statistically significant (*p* = 0.10 for both time points).

Moreover, there were significant differences between the two groups in terms of CAL at both the 4th and 8th weeks (*p* < 0.001 and *p* < 0.001, respectively) and a greater reduction in GI at the 4th week (*p* = 0.01) (Table 2).

## 4 Discussion

Periodontal disease is primarily caused by microbial biofilms, making their removal the central goal of periodontal treatment. Although SRP remains the foundation of nonsurgical therapy, achieving complete debridement, especially in deep areas, can be challenging. To overcome this limitation, adjunctive antiseptic irrigants such as chlorhexidine, hydrogen peroxide, and essential oils have been explored. Among these, ozonated water has gained attention for its strong antimicrobial and anti‐inflammatory effects. Its topical application into periodontal pockets, through methods like syringe irrigation, has shown promise in enhancing biofilm reduction (Al Habashneh et al. 2015; Katti and Chava 2013; Yılmaz et al. 2013; Hayakumo et al. 2013; Kshitish and Laxman 2010; Uraz et al. 2019; Estrela et al. 2007).

In this clinical trial, we explored the application of ozonated water as an adjunctive antiseptic agent in the nonsurgical treatment of patients with periodontal disease. Patients were randomly assigned to two groups of 15 individuals each ‐ the test group received ozonated water, while the control group was treated with tap water. This innovative design ensured that the test group benefitted from prolonged contact duration between ozonated water and microorganisms residing in the periodontal pockets, thus potentiating ozone's antimicrobial effects.

Since the roughness on the root surface caused by ultrasonic scalers can affect plaque retention, the treatment endpoint was determined when the root surface felt completely smooth upon tactile sensation using a periodontal explorer.

The findings showed a significant decrease in clinical parameters, including CAL, PPD, and GI, at both the 4th and 8th weeks in both the test and control groups. This reduction reflects the known effectiveness of SRP in improving periodontal health. Although improvements were observed in both groups, the reduction in these parameters was more pronounced in the test group receiving ozonated water. In particular, greater improvements in CAL at both follow‐up points and in GI at the 4th week were statistically significant, suggesting an additional benefit from the adjunctive use of ozonated water compared to SRP alone. These results are consistent with previous studies evaluating the adjunctive effects of ozonated water in periodontal therapy (Al Habashneh et al. 2015).

Regarding the effect of SRP using ozonated water on PPD, a decrease was observed in both groups at 4 weeks and 8 weeks. Although this decrease was more pronounced in the test group compared to the control group, the difference between the groups was not significant. This finding aligns with previous studies, likely due to the slow progression of clinical parameters following nonsurgical periodontal treatment. Studies by Badersten and Huth indicated that approximately one to three months are necessary for a proper evaluation of the progress of clinical parameters after mechanical debridement (Phase I of periodontal treatments) (Badersten et al. 1984).

A reduction in CAL was observed in both groups at the 4‐week and 8‐week marks as a result of SRP with ozonated water. This reduction was significantly higher in the test group compared to the control group. Al Habashneh examined the effect of SRP combined with irrigation using ozonated water and sterilized water on 41 patients with chronic periodontitis. The results demonstrated that, after 3 months, all clinical parameters—including PPD, bleeding on probing (BOP), CAL, and plaque index (PI)—were significantly decreased in both groups. However, the study found no statistically significant difference between the groups, indicating that ozonated water did not show superior clinical outcomes compared to the control treatment (sterilized water) (Al Habashneh et al. 2015).

Additionally, Skurska studied 52 patients with chronic and aggressive periodontitis to compare the efficacy of SRP combined with topical application of ozonated water to SRP alone. The results indicated a significant reduction in clinical parameters, including PPD, PI, CAL, and BOP, in both groups after 2 months, with no significant difference between the groups for any parameter over follow‐up visits (Skurska et al. 2010).

In this study, no statistically significant difference was observed between the groups in terms of PPD during follow‐up visits. However, although a statistically significant difference was found for CAL, the magnitude of this difference was minimal and of limited clinical relevance. A detailed examination of the data revealed that in certain sites for some patients, PPD either remained unchanged. Nonetheless, the mean PPD values in both groups remained below 3 mm throughout the study period, which is generally considered a clinically healthy or controlled periodontal condition. This could be attributed to factors causing mild gingival enlargement in those regions, such as poor adherence to oral hygiene during follow‐up periods, dental crowding, or having inappropriate crowns or restorations that made it difficult for the patient to access those areas. This gingival enlargement might contribute to the nonsignificant differences observed in PPD.

The effect of SRP with ozonated water on GI resembles the findings for CAL. Both groups experienced a significant reduction in GI after 4 and 8 weeks. Specifically, at the 4‐week mark, the reduction in GI was significantly greater in the test group compared to the control group. This observation aligns with a study conducted by Isaac on 30 patients with mild to moderate periodontitis. In Isaac's study, the clinical parameters were assessed 4 weeks posttreatment, and it was found that the GI significantly decreased in the group receiving SRP combined with ozonated water irrigation (Issac and John 2015). The observed reduction in GI may be attributed to the anti‐inflammatory properties of ozone. Supporting this, Dhingtra's study on 15 patients with gingival inflammation during orthodontic treatments demonstrated that subgingival irrigation with ozonated water increased gingival crevicular fluid (GCF) lactate dehydrogenase (LDH) activity after 4 weeks, thereby reducing gingival inflammation (Nogales et al. 2008).

### 4.1 Study Limitations

This study has several limitations that should be considered when interpreting the results. First, the relatively small sample size (*n* = 29) may limit the generalizability of the findings to broader populations. Second, the follow‐up period of 8 weeks may not fully capture the long‐term clinical outcomes of periodontal therapy with ozonated water. Longer‐term studies are needed to confirm the sustained benefits observed in the short term. Third, microbiological assessments were not performed to directly measure the bacterial load reduction; therefore, the antimicrobial effect of ozonated water was inferred indirectly through clinical parameters. Future studies incorporating microbiological and inflammatory biomarkers could provide a more comprehensive understanding of the therapeutic effects of ozonated water in periodontal treatment. And finally, in this study, periodontal measurements—including PPD and CAL—were performed at the mesiobuccal, midbuccal, and distobuccal sites only, with no measurements taken on the lingual or palatal surfaces. This site selection was intentional and based on the need to ensure standardization, reproducibility, and ease of access during clinical examinations. Buccal sites generally provide more consistent anatomical landmarks and are more accessible for accurate probing, reducing variability across time points and between patients. However, we acknowledge this as a limitation of the study, as excluding lingual and palatal sites may have led to a partial representation of the overall periodontal condition. Future studies with full‐mouth six‐site measurements per tooth would provide a more comprehensive assessment.

## 5 Conclusions

The present study demonstrated that both the test group (ozonated water) and the control group (tap water) showed significant clinical improvement in PPD, CAL, and GI following nonsurgical periodontal therapy. While no statistically significant difference in PPD reduction was observed between groups, the test group showed significantly greater improvement in CAL at both the 4‐week and 8‐week follow‐up points, and a greater reduction in GI at the 4‐week mark.

These findings suggest that the adjunctive use of ozonated water may contribute to improved clinical parameters, particularly in terms of attachment level gain and reduction of gingival inflammation. However, the magnitude of these improvements was small, and their clinical relevance may be of modest clinical relevance.

Nevertheless, further research with larger sample sizes and longer follow‐up is needed to confirm these outcomes and evaluate long‐term effectiveness.

## Author Contributions

Mohadese Yazdanpanahbahabadi conceived the study, designed the methodology, and performed the clinical intervention of the study. Mojan Ghafouri collected the data and performed the analysis. Hamed Hosseinkazemi drafted the manuscript. Majid Fereidooni contributed to patient allocation and designed the treatment.

## Conflicts of Interest

The authors declare no conflicts of interest.

## Data Availability

The data sets analyzed during the current study are available from the corresponding author on reasonable request. Data is available on request from the authors.
